# Minimum standards for reporting outcomes of surgery in urogynaecology

**DOI:** 10.1007/s00192-020-04575-z

**Published:** 2020-10-28

**Authors:** Philip Toozs-Hobson, Fiona Bach, J. Oliver Daly, Niels Klarskov

**Affiliations:** 1 Birmingham Womens and Children’s NHS Foundation Trust, Birmingham, UK; 2 Christchurch Women’s Hospital, Christchurch, New Zealand; 3 Department of Obstetrics and Gynaecology, Sunshine Hospital, Western Health, Melbourne, Victoria Australia; 4 Department of Epidemiology and Preventive Medicine, Monash University, Melbourne, Victoria Australia; 5 Department of Obstetrics and Gynecology, Herlev and Gentofte Hospital, Herlev, Denmark; 6 Faculty of Health and Medical Sciences, University of Copenhagen, Copenhagen, Denmark

**Keywords:** Surgical database surgical outcome measurement

## Abstract

**Introduction and hypothesis:**

The IUGA special interest group (SIG) identified a need for a minimum data set (MDS) to inform outcome measurements to be included and simplify data capture and standardise reporting for data collection systems. To define a minimum data set for urogynaecological surgical registries.

**Methods:**

Existing registries provide an inventory of items. A modified Delphi approach was used to identify a MDS. At each stage reviewers ranked data points and used free text to comment. The rating used a scale of 0–10 at each review and a traffic light system rated the scores as desirable, highly desirable and mandatory. The scores were collated and reported back to clinicians prior to the further rounds. Outliers were highlighted and reviewers re-assessed prior to repeating the process. A comparison of the MDS was made with published outcomes.

**Results:**

Reviewers were from the outcome SIG with emphasis on widespread representation. Fifteen clinicians from eight countries were involved. Four reviewers dissected the existing databases. Eighty data points were considered in four categories, background, preoperative, intraoperative and postoperative. Consensus was reached by the third round. Two points were added on review (date of surgery and urodynamics). Three background points, five preoperative points, seven intraoperative points and nine postoperative points were identified giving 24 minimum data points in the final recommendation.

**Conclusions:**

An MDS has been developed for urogynaecological surgical registries. These should be mandatory points which then allow larger varying points to be assessed. These points correspond well to data points used in published papers from established databases.

## Background

Interest in measuring outcomes is increasing. The International Consortium for Health Outcomes Measurement (ICHOM) [1] was formed based on the principles set out by Porter and Teisberg in their book Re-defining Healthcare [2]. In urogynaecology the Austrian TVT registry probably represents the first early attempt at collecting systematic data [3, 4]. In 2006 the first national prospective databases emerged in Denmark and the UK [5, 6]. The Danish DUGAbase was established by urogynecologists to describe the extent and quality of urogynaecological operations as well as to monitor new surgical techniques and implants. The database is now financed by the public health care authorities and a report is published yearly with data from each department describing 15 indicators. Data from the database can be supplied on request for scientific purposes.

These resources have over time been invaluable in providing insight into practice and outcomes, as was highlighted by the publication of the national audit on stress incontinence. Denmark mandated their database so that it could link to other national databases using a unique patient reference. Other databases such as the AUGS AQUIRE database [7] and the Australasian Pelvic Floor Procedure Registry [8] are in the process of developing modules, and there is a clear need to define a MDS to leverage the value of large data sets.

The International Urogynecological Association (IUGA) database was established in 2016 by Paul Moran after a member’s survey suggested there was widespread interest in having access to a database for personal audit. This in itself was based on a cut-down version of the British Society of Urogynaecology (BSUG) database and represented the first attempt to simplify data collection. The rationale for using a database has been set out in the International Urogynaecology Journal (IUJ) by both Abdelrahman et al. [9] and Toozs-Hobson et al. [10]. Commonly cited obstacles to using databases revolve around the time and effort combined with no additional resources to complete data entry at any point.

IUGA have over recent years established a number of specialist interest groups (SIG) with the surgical database being established in 2019 [11]. One of the first objectives of this group was to focus on the identification of important data items and outcomes while reducing the burden of data entry. As such we sought to identify key items that should be universal in all urogynaecological databases. In the principle of ICHOM this MDS would allow base data to be routinely collected with the collection of additional data items enabling the answering of particular questions by the collection of large volumes of data. For example, at a national level, there should be sufficient surgical volumes to collect data on 50,000 cases looking specifically at mesh complications of tape to give the true incidence, relationship to age and BMI and then allow for linkage with secondary data to look at re-operation rates.

### Objective

To define a minimum data set for inclusion in registries as using existing reference data sets for clinicians in clinical practice.

### Methods

Reviewers were invited from within the SIG with additional experts invited to ensure widespread representation. Members of the IUGA surgical database SIG identified established databases and registries for comparison. Full data sets of these registries were examined and duplicate data points removed by reviewers. There was no representation within the SIG from other areas (e.g. Central and South America and Africa). The combined data set was then used for the initial review as baseline. This process used a modified Delphi approach [12, 13] with the aim of reaching consensus on the most important items. During the rounds the reviewers were able to suggest additional items not included from the initial scoping exercise. The initial aim, aspirationally and arbitrarily, was to identify a reduced data set of 20 points, as this was deemed an acceptable additional burden.

At each stage reviewers were invited to first score the importance (0–10). The rating gave a scale of 0–10 at each review and a traffic light system rated the scores as required, desirable, highly desirable and mandatory. Reviewers then also identified their most important ten items, listing one to ten for importance. The scores were then collated prior to the second round. This review identified outliers which were then highlighted based on distribution of anonomysed responses and then recirculated to reviewers. Items that scored poorly were rejected to reduce the burden of assessment. Responses were then re-analysed as after the first round to produce a similar document of modified response prior to being re-assessed. The aim was to repeat the process until consensus was reached. At each stage there were free text boxes available to allow comment and add additional points for consideration. The process was expected to have three rounds to allow opportunity for reviewers to consider the results.

### Results

Reviewers included 15 clinicians from 8 countries giving widespread representation from Australasia, North America and Europe. Four reviewers volunteered to dissect the existing data sets including the IUGA database, BSUG database and DUGAbase. Eighty data points were considered. These were broken into four categories of background data, preoperative data, intraoperative and postoperative data points. Consensus was achieved by the third round. Two points were identified during the first review that had not been included in the first round which were added on review (date of surgery and urodynamics) as these were thought to be omissions on review.

After two rounds 24 points were deemed essential as the minimum data set. These consisted of three background points, five preoperative points, seven intraoperative points and nine postoperative points which were included in the final recommendation. These points are included in Table 1 below.

**Table 1:** Key data points

Time point	Data points
Background	1. Age 2. BMI 3. Date of surgery
Preoperative	4. Previous incontinence surgery 5. Incontinence symptoms 6. Previous POP surgery 7. Prolapse symptoms 8. Urodynamics (whether performed and if so the result)
Intraoperative	9. Grade of operator 10. Procedure and type 11. Prosthesis type (if used) 12. Intraoperative complications *- Bleeding > 500 ml* *- Drain* *- Bowel injury* *- Ureteric injury* *- Bladder injury* *- Urethral injury* 13. Intraoperative transfusion 14. Return to theatre (72 h) 15. Concurrent surgery
Postoperative (defined time 6 weeks/6 months/1 year)	16. Change in urgency 17. Recurrence 18. Catheterisation > 10 days 19. Complication 20. GII incontinence 21. GII prolapse 22. POPQ, if face to face 23. Death 24. Return to hospital < 30 days

The data points highlighted the theme that was to be collected. There was no attempt to inform the choice of exact tools. For example, incontinence symptoms would include stress, urgency and mixed. The aim was to give a high-level overview rather than detailed in-depth breakdown. Prolapse symptoms may include a sensation of bulge and backache. Likewise, urodynamics should include whether evaluation was performed (yes/no) and may be a simple descriptive such as urodynamic stress incontinence, detrusor overactivity, mixed urinary incontinence and voiding difficulties. Procedure type refers to primary or repeat procedures.

Postoperatively, the main outcome should be defined using a 7-point scale of very much worse, much worse, slightly worse, no change, slightly better, much better and very much better, like in the Patient Global Impression of Improvement (PGI-I).

## Discussion

Databases represent a potentially powerful tool to continuely improve practice. Whilst randomised controlled trials represent the gold standard of comparing two treatments, they do not answer all the questions and larger data sets may provide a degree of granularity to more subtle areas of real-life practice with a pragmatic approach. Routine collection of data should not be burdensome and is inexpensive (compared to a RCT). Whilst this is the list that was developed as part of the Delphi process, we accept that it represents a minimum list and may not be the perfect list. The included items reflect well on currently published series and it was the list that was agreed on by the clinicians involved. There are potential problems with recording the postoperative POPQ as many clinicians do not see their patients physically in the clinic postoperatively and this has become more commonplace following COVID-19 whereas the Delphi process was prior to this so may need to be moderated. For an individual clinician, the purpose of collecting data is to facilitate audit of their patient’s outcomes. However, a wider co-operative approach collating the outcomes of a consortium of clinicians into large data sets provides the ability to look at outcomes in a wider context to analyse and report on common, uncommon and rare events. The benefits of having routine outcome data both individually and collectively will drive improvements in management. The individual clinician may gain deeper insight into their practice and be able to see trends or potential variations before others and this gives the opportunity to adjust practice early. The key point is to collect the necessary data prospectively to inform this process.

Another point of contention is ownership of and access to the data. With a mandatory database this is clearly the governing body that uses the data in accordance with its data access and reporting obligations. With a voluntary database the data should be owned by the individual and the database should have an agreement in place to cover this. Regarding the IUGA database, this is accomplished by complete anonymisation of the data through a unique reference for the patient to comply with international health data privacy laws.

The aim of our process was to standardise the core data items and simplify the quantity of data required in a urogynaecological surgical database to make large-scale data collection feasible. Just as with research, the prospective identification of critical data points is important. Currently published data from the established databases use only a fraction of the recorded data in publications. As such the benefits of extensive data collection may be lost by the administrative burden resulting in not collecting data. The advantage of having a minimum data set is that it dramatically reduces the data entry required, but still allows interesting comparisons, e.g. age alone as a predictor of outcome from retropubic tapes [14]. The aim of this as a minimum data set would be to allow rapid data assimilation, with reduced additional resources required. It does not preclude the use of larger data sets, but prospectively would allow specific questions to be posed and changing additional information to be gathered, e.g. this year we would like all the urodynamic data. Once a question has been answered the data points may become redundant and be replaced by those that answer a different question. The aim of this study is therefore to encourage data collection and contribute to the wider understanding. In time, it is hoped this can be developed to focus on specific areas to answer specific questions. Such an approach requires a much broader and more universal collection of data and it is hoped that IUGA and the SIG in particular may help lead this process.

### Conclusions

This article presents the IUGA Outcomes SIG recommendation for an MDS. This represents the SIG’s collective view of the minimum data points to be noted and should be suitable for mandatory inclusion in any registry. The MDS was defined during a modified Delphi process with worldwide representation. The aim of this process was to standardise and simplify the data collected to ease the individual clinician burden in contributing to large data sets. These core data items can be used as mandatory points which then allow larger varying points to be assessed either routinely or by exception depending on research or audit questions. These points correspond well to data points used in published papers from established databases. The IUGA database allows members to use this MDS.
